# Challenges and strategies for general practitioners diagnosing serious infections in older adults: a UK qualitative interview study

**DOI:** 10.1186/s12875-019-0941-8

**Published:** 2019-04-26

**Authors:** Sara McKelvie, Abigail Moore, Caroline Croxson, Daniel S. Lasserson, Gail N. Hayward

**Affiliations:** 1 Nuffield Department of Primary Care Health Sciences, University of Oxford, Radcliffe Primary Care Building, Oxford, OX2 6GG UK; 2 Institute of Applied Health Research, University of Birmingham, Birmingham, UK; 3 Department of Elderly Care, Queen Elizabeth Hospital Birmingham, University Hospitals Birmingham NHS Foundation Trust, Birmingham, UK

**Keywords:** Clinical decision-making, Geriatric, General practitioners, Infection, Older people, Primary health care, Qualitative research

## Abstract

**Background:**

Serious infections in older people are associated with unplanned hospital admissions and high mortality. Recognising the presence of a serious infection and making an accurate diagnosis are important challenges for General Practice. This study aimed to explore the issues UK GPs face when diagnosing serious infections in older patients.

**Methods:**

Qualitative study using semi-structured interviews. 28 GPs from 27 practices were purposively sampled from across the UK to achieve maximum variation in terms of GP role, experience and practice population. Interviews began by asking participants to describe recent or memorable cases where they had assessed older patients with suspected serious infections. Additional questions from the topic guide were used to explore the challenges further. Interview transcripts were coded and analysed using a modified framework approach.

**Results:**

Diagnosing serious infection in older adults was perceived to be challenging by participating GPs and the diagnosis was often uncertain. Contributing factors included patient complexity, atypical presentations, as well as a lack of knowledge of patients due to a loss in continuity. Diagnostic challenges were present at each stage of the patient assessment. Scoring systems were mainly used as communication tools. Investigations were sometimes used to resolve diagnostic uncertainty, but availability and speed of result limited their practical use. Clear safety-net plans shared with patients and their families helped GPs manage ongoing uncertainty.

**Conclusions:**

Diagnostic challenges are present throughout the assessment of an older adult with a serious infection in primary care. Supporting GPs to provide continuity of care may improve the recognition and developing point of care testing for use in community settings may reduce diagnostic uncertainty.

## Introduction

Serious infections diagnosed in adults aged 65 and over are a leading cause of hospital admissions and are associated with a high mortality [1, 2]. In the UK, an estimated 58% of older patients with a same-day diagnosis of community acquired pneumonia (CAP) in primary care are hospitalized [3]. Hospitalization for older adults with serious infections is associated with persistent functional decline and high mortality [3, 4]. Detecting the presence of serious infections is often difficult due to atypical presentations and non-specific signs of infection in older patients [1, 2]. Therefore, distinguishing which older patients have serious infections remains an important challenge for clinicians, particularly those working in primary care.

In the UK, General Practitioners (GPs) play a central role in identifying and managing infection in older adults. During working hours, GPs are responsible for assessing patients with acute problems within the catchment area of their practice. Outside of this time, patients can also access Out-Of-Hours (OOH) GPs, but these work within an urgent care service and do not necessarily know the patient or have access to their medical record.

Following a diagnosis of infection in older adults, GPs also face the problem of assessing severity and predicting prognosis. Scoring systems such as the Pneumonia Severity Index (PSI) and CRB65 score (both for assessing the severity of community acquired pneumonia from simple clinical observation) have been recommended to aid primary care prognostic decisions [5]. However, these scores were developed to predict mortality during a hospital assessment [6], and limitations apply when using these scores for older patients in the community, as they were not developed for, or validated in, this population and do not include clinical co-morbidities or social factors [7].

Given the difficulty of diagnosing and predicting prognosis for older patients with serious infections, it is critical that this complex clinical decision-making is understood, as there are implications for patient safety (avoiding complications of infection), antimicrobial stewardship (over-use of antibiotics) and managing demand for acute care. We used a qualitative approach to explore in depth the issues for UK GPs diagnosing serious infections in older adults and the techniques used to meet these challenges.

## Methods

The study team compromised a mix of clinicians and non-clinicians, including GPs at different stages of their careers and ambulatory care/care of older people expertise. All authors involved in study design, data collection and analysis have training in qualitative methods and were supported throughout by CC, a senior qualitative researcher and social scientist.

### Recruitment

UK GPs were recruited via email invitations sent through Clinical Commissioning Groups, Royal College of General Practitioners and RuralGP.com mailing lists, which included a brief description of the study. Those who participated were offered a small reimbursement for their time. Purposive sampling amongst GPs who answered advertisements was used to ensure variation in experience, role, practice location and practice size. Recruitment ended once the research team agreed that data saturation had been reached – based on the fact that no amendments had been made to the topic guide, no new codes had been added and no new significant themes had emerged for several consecutive interviews. Ethical approval was granted by the University of Oxford Medical Sciences Interdivisional Research Ethics Committee MS-IDREC-C1–2015-054.

### Data collection

Interviews were conducted by a female GP clinical lecturer (GH) and a female academic GP registrar (AM). Participants were asked to describe recent or memorable cases where they had diagnosed an older person (aged over 70 years) with a serious infection, prompted by the topic guide (Table 1). The topic guide was based on existing literature [8, 9] and research team expertise, and evolved during the study with discussion of the emerging themes by the research team. Four participants were known to the interviewers beforehand in a professional capacity. All participants were made aware of the aims of the research. The interviews were carried out face-to-face [4] or over the phone [24] and lasted up to 40 min in duration. Audio-recordings were transcribed verbatim.

**Table 1:** Flexible topic guide for the interviews with 28 GPs

Questions making up the flexible topic guide	
Describe the clinical case and context.	
Tell me about the signs and symptoms you elicited and why? How did you interpret the findings?	
What is your experience of point-of-care testing?	
What is your experience of other investigations?	
What were the views of the patient or patient’s carers?	
Please summarise your decision-making process regarding admission.	
What are the potential benefits and disadvantages of staying at home compared to an admission?	
What options are available to you in your area for further investigation and inpatient or out of hospital care, and what are your thoughts on these? How do you access these?	
What is your experience of advance care plans?	
What are your views on medicolegal issues surrounding admission?	
What are your views on use of resources?	
What advice would you give to other GPs about diagnosing infection in older patients and deciding whether to admit?	

### Data analysis

A modified framework approach to analysis followed the key steps of transcription, familiarisation, coding, developing an analytical framework, charting of themes and interpretation [10]. The coding framework was derived from the topic guide and refined after initial double coding of transcripts by AM and GH and discussion amongst the whole research team. Subsequent coding and analyses were completed by AM and SM, with further group discussion to resolve differences and combine or remove codes where appropriate. Group discussions allowed reflexivity amongst the team. Charting was used to organise and display material relating to the emerging themes [11]. Charts were shared amongst the whole research team and generated discussion. NVivo (version 10) was used to facilitate coding but was not used for charting.

Constant comparison of the interviews ensured that themes and concepts were grounded in the data [12]. The research team took an iterative stance from the outset – combining early analysis with ongoing data collection. This helped to shape future data collection, including helping decide where sample needed broadening (eg. different geographical locations) and ensuring that the coding framework continued to fit new data.

This article focuses on the challenges and strategies that GPs use when making diagnostic and prognostic decisions for older people with suspected serious infections. Antibiotic use in older adults and decisions regarding hospital admission are the subject of separate articles [13, 14].

## Results

Thirty eight GPs responded initially to advertisements. After further communication, 28 GPs from 27 different practices consented to take part in the study and were interviewed. The participants varied in their experience level, GP roles and practice populations (Table 2).

**Table 2:** Characteristics of the 28 GPs interviewed

GP characteristics (*n* = 26)	
Sex	
Male	13
Female	13
GP role (*n* = 25)	
Partner	12
Salaried	6
Locum	3
OOH	3
Years as a GP	
1–5	7
6–10	5
11–15	4
16–20	2
> = 21	8
Practice characteristics (n=24)	
Location (n = 24)	
Rural	7
Suburban	5
Urban	3
Mixed	8
List size (*n* = 19)	
≤ 5000 (small)	6
5001–10,000 (medium)	11
10,001–15,000 (large)	3
Number aged > 75 (*n* = 14)	
≤ 500	7
501–1000	3
> 1001	4

Cases discussed were predominantly chest infection, urine infection, cellulitis or infection of unknown source. However, the case mix also included rarer diagnoses such as discitis, appendicitis, joint infection, gastrointestinal infection and candidiasis (Table 3).

**Table 3:** Cases discussed during interviews with 28 GPs

Cases	Frequency discussed
Chest infection	28
UTI	11
Infection of unknown source	11
Cellulitis	9
IECOPD	2
Sepsis	2
Appendicitis	1
Aspiration pneumonia	1
*C. difficile*	1
Candidiasis	1
Discitis	1
Diverticulitis	1
Gastroenteritis	1
Joint infection	1
Sore throat	1

The main themes developed from the data are summarised in Table 4.

**Table 4:** Major themes and subthemes emerging from interviews with 28 GPs

Major theme	Subthemes	
Challenges leading to diagnostic uncertainty	Patient complexity	Multi-morbidity, polypharmacy, social needs
	Atypical presentation	Non-specific symptoms and signs
	Knowledge of patient	GP workload, continuity of patient care
Approaches to recognising the suspected serious infection in older adults	History taking	Recognition of non-specific symptoms including confusion, falls, incontinence, unsteadiness, lethargy and vomiting Change from baseline Collateral history from relatives and carers, reviewing notes
	Physical examination	Standardised systematic approach including temperature, respiratory rate, pulse, blood pressure and oxygen saturation Exam findings often not considered trustworthy, role of gut feeling
	Scoring systems	Judge disease severity, communication to secondary care
Strategies to manage diagnostic uncertainty	Investigations	Varied levels of trust in different tests, ease of access
	Safety netting	Safety net through third party, shared decision making

### Theme 1: challenges leading to diagnostic uncertainty

Diagnosing a serious infection in older adults was recognised by all the participating GPs to be a difficult task, caused by the interplay of various factors, and resulting in diagnostic uncertainty.

*“It’s really, really hard. I think it’s an absolute minefield actually. I think the main things are actually you trying to cope with your own uncertainty and knowing often that elderly people are different and unpredictable and that you won’t always have a diagnosis…”* (GP27, female partner, 6–10 years’ experience, large urban practice)

#### Patient complexity

GPs described how assessing acute illness in older people was frequently confounded by the background of multiple complex medical and social needs. GPs talked about how multi-morbidity and polypharmacy could affect their interpretation of their assessment, and how co-morbidities might impact the severity of illness.

*“When it comes to other measures, the tachycardia which we would measure isn’t a very good thing if someone’s on beta blockers, for instance, or if they have a pacemaker.”* (GP16, male partner, 6–10 years’ experience, small suburban practice)

#### Atypical presentation

Most GPs recognised the challenge of the atypical presentation of older patients with serious infections. Many believed that classic signs of infections could not be relied upon (see also Examination, below), and that the non-specific symptoms might also be indicative of an alternative diagnosis.

*“The first sign might be the person’s off legs, falls, or they’re getting confused and then the difficulty there is that a chronic confusion or an acute confusion or acute and chronic and are they falling because they have postural hypotension or their place isn’t safe, or are they falling because they’ve got a urine infection?”* (GP16, male partner, 6–10 years’ experience, small suburban practice).

#### Knowledge of the patient

GPs cited the lack of sufficient background information or perceived lack of continuity as a particular challenge in this context. Locum GPs (GP2, 6 & 12) or OOH GPs (GPs 21,22 & 26) were very aware of the reduced information that they had in comparison with the patients’ regular doctor.

*“I think it depends on how well you know the person, because if you know someone well, then you’ll know when they’ve changed. So whether they’re talking the same way, whether they’re more confused, their general state, how they’re getting around, how mobile they are, that’s one side of it, but that depends on knowing people well. If you meet someone for the first time, it’s harder to assess that.”* (GP16, male partner, 6–10 years’ experience, small suburban practice).

A reduction in continuity of care was attributed by some participants to an increase in part-time working, with different doctors following up the same patient. Increases in GP workload, with other demands on time and a reduction in capacity for home visiting, was also described as affecting the ability to really know older patients.

“*I think the big issue for me…is the continuity and that’s been the big change over the years, that GPs are mostly part-time and how do you manage handing over and I think we need to have a more formal buddy system that you can say my colleague will be picking this up tomorrow if I’m not there, I think that’s going to be a challenge for the future.”* (GP17, male partner, ≥21 years’ experience, large suburban practice)

### Theme 2: approaches to recognising the suspected serious infection in older adults

#### History taking

Careful history taking was described as the most important way to obtain diagnostic information. Sometimes the diagnosis was clear from the history. However, more often the history instead revealed non-specific symptoms including confusion, falls, incontinence, unsteadiness, lethargy and vomiting. Changes in functional ability were described as important in judging disease severity.

*“So if they’ve gone from going down Tesco’s to now actually not being able to leave the chair that would be quite an extreme and rapid, you know, decline in their function”* (GP7, male locum GP, 1–5 years’ experience, medium sized suburban practice).

When patients were known to the GP, an assessment of their change from baseline was discussed. GPs described patients as not being their usual self and this was synonymous with illness. Change from baseline could be identified by patients, relatives and carers.

Frequently GPs outlined cases where the history had been difficult to obtain, for example, due to the presence of delirium or dementia. In these cases, participants described using several different strategies; obtaining a contemporaneous collateral history from family members or carers, relying on their physical examination findings or looking for relevant entries in the medical notes.

*“There was very little history available from the patient because he was only responding to voice, he was slightly confused. So the initial assessment was really a physical assessment, having taken a history from the family and as much as possible from the notes that we had available at that time which were very few, very scant”* (GP21, Male OOH GP, 16–20 years’ experience urban location).

#### Physical examination

Firstly, participants decided whether patients ‘looked unwell’. This was described as a combination of pattern recognition and experience. Clinicians trusted their ‘gut instinct’ and described it as an important feeling to be used in conjunction with the medical assessment.

*“I think as doctors you do it all the time, in making that sort of judgement of whether they are well or not. It doesn’t always work, and sometimes you don’t have that, you haven’t really got a clue sometimes what’s going on with some patients, but I think certainly the extremes, you can tell who’s really well and you can tell who’s really ill”* (GP12, female locum GP, 6–10 years’ experience, large mixed practice).

GPs then described performing a systematic examination by measuring vital signs (temperature, respiratory rate, pulse, blood pressure and oxygen saturation) as well as a physical examination to assess the older person with suspected infection. When describing the examination, GPs often considered the parallels between assessing older adults and paediatric patients and the resultant need to be thorough.

*“Elderly patients, it’s almost like they’re children…they don’t present with obvious signs or symptoms”* (GP6, female locum GP, 1–5 years’ experience, small urban practice).

In contrast to gut instinct, vital signs could not always be trusted in older patients. For example, although a high temperature would confirm infection to a GP, a low or normal temperature was not reassuring.

*“If I think somebody’s poorly, but they’ve got a normal temperature, that doesn’t stop me from still thinking that they’re poorly, if that makes sense. So I take it, and if it’s high and I think they’ve got an infection then it kind of, helps me to be more convinced, but I wouldn’t not act or not start treatment on somebody who I thought had an infection just because their temperature was normal on my thermometer, because I don’t think it’s 100% reliable”* (GP15, female GP salaried GP, 16–20 years’ experience, suburban practice).

Respiratory rate was discussed as a reliable marker of acute infection, although breathlessness was used by some GPs as a surrogate.

*“I’m afraid I have to confess, I’m never great at objectively measuring respiratory rate, I tend to go on whether they’re panting when they come in the room”* (GP24, male partner, ≥21 years’ experience, suburban practice).

The pulse oximeter was considered a standard piece of equipment and oxygen saturation was commonly measured. However, the pulse oximeter was only felt to be useful in patients where the baseline level was known.

*“Do I find saturations reassuring, sometimes, I find it reassuring to explain to the patient, but they also can be fairly unreliable in my experience”* (GP2, female locum GP, 1–5 years’ experience, urban location).

If, at the end of the examination, no concerning clinical signs were revealed and the GP was uncertain of the diagnosis or had a gut instinct that something was wrong, this was a prompt to clinically re-evaluate the patient.

*“And if you’re getting everything coming back normal and someone is still not right then there’s probably something else going on that might need to be looked at”* (GP7, male locum/salaried GP, 1–5 years’ experience, medium-sized suburban practice).

#### Scoring systems

Scoring systems (early warning scores or CRB65) were used by some GPs. In practice, these scores were mostly used to judge disease severity and communicate urgency to secondary care teams. For example, the CRB65 score for CAP was familiar to many GPs and this score was used as a negotiating tool when discussing hospital admission.

*“For a pneumonia I do the CRB65, I have probably made my decision without formally doing the scoring system and I probably use the scoring system more either as backup if I’m trying to admit the patient say, well look, their CRB65 is this”* (GP25, female partner, 1–5 years’ experience, medium-sized suburban practice).

Others were unsure of the validity of scoring systems in the community and were cautious about score interpretation. GPs often felt scores oversimplified the assessment of older patients. Some GPs described feeling overwhelmed by the number of different scoring systems but a few GPs explained that scores could be useful for inexperienced clinicians.

### Theme 3: strategies to manage diagnostic uncertainty

#### Investigations

Further investigations could be arranged where diagnostic uncertainty remained after the initial history and examination. Investigations ordered could include urine tests, blood tests or chest x-rays. Like the physical signs, investigations were described according to their perceived reliability. Tests were ordered if GPs felt that the results could change the patient’s clinical course but clinicians expressed doubts about reliability.

*“There are things that are useful that may come back within 24 hours that will help you decide, for example, a raised white cell count, raised CRP, positive urinary infection, I’d just say, if there was an element of uncertainty, then I would be checking some tests”* (GP1, female partner, ≥21 years’ experience, medium-sized suburban practice).

Urine dipstick testing was widely reported but interpretation was often problematic. Some GPs considered dipstick testing helpful if the patient was non-specifically unwell or when associated with other symptoms (abdominal or back pain). Urine dipsticks in catheterised patients, or results showing protein or white cells were considered unhelpful for diagnosis. Several GPs felt the presence of nitrites in the urine was almost synonymous with a diagnosis of a urinary tract infection. Other participants reported that urine dipsticks might demonstrate false negative results and so could not rule out urine infections.

*“I always do one [a dipstick] but I have had some cases in the past where the MSU [urine culture] has come back positive and the dipstick hasn’t shown much*” (GP9, female locum GP, 1–5 years’ experience, medium-sized practice).

Urine cultures were described as useful if bacterial growth was detected, and to target antibiotic treatment. However, drawbacks reported were time delays waiting for results and findings showing asymptomatic bacteriuria.

*“They’re [urine cultures] a bit hit and miss, aren’t they? They’re fairly unreliable I think, ‘cos there are lots of elderly people who will have abnormal dips with their urine, and even abnormal cultures that are just colonised rather than infected. So sometimes that’s not definitely helpful. I guess you have to look at the whole picture, don’t you?”* (GP15, female salaried GP, 16–20 years’ experience, suburban practice).

The practicalities involved in obtaining some tests limited their use. Locum GPs were particularly aware of how test availability varied depending on their setting. Some GPs also felt that waiting for test results would delay decision-making and treatment.

*“If I wanted bloods it would be like a domiciliary phlebotomist which, you know, would take a day or two and then a day or two for the results to come through, so I almost find myself making decisions without adding that into the equation a lot of the time, you know, it’s either does this patient need admitting to have those results right now or can we just treat in the community and see how it goes.”* (GP5, female salaried GP, 6–10 years’ experience, medium-sized urban practice).

For most GPs interviewed, radiographic findings on chest x-rays inspired greater belief than examination findings, but x-rays were difficult to obtain. Rural GPs would consider whether obtaining a chest x-ray would significantly impact on their management due to the associated transport requirement (patients might have to travel some distance to get to a facility that offered this test).

Most GPs had little experience in using point of care (POC) blood tests (tests performed at the bedside not requiring laboratory analysis with rapid results). There was a described preference for relying on clinical signs.

*“I’d still rather go on my clinical signs really, I think they’re the best reliable indicators for me as to how am I gonna manage this patient. I mean because if someone had a CRP of 200 or something and you’re thinking they’re septic they, you’ll have some signs there that they will be septic”* (GP9, female salaried, 1–5 years’ experience, medium-sized suburban practice).

GPs were split on the potential use of POC testing in primary care. Many felt that POC tests could provide useful information if they were easy, reliable and cheap to use. Others wished for more evidence of patient benefit before changing their clinical practice. These views were not related to where the GP worked; those working in more acute settings (OOH and locum work) did not express any preference for POC testing.

#### Safety-netting

Participants commonly described an anxiety that deteriorating older people were less likely to seek further medical assistance.

*“I think younger people are far more likely to just call another doctor later that day for a second opinion. I think the elderly are much more prepared to go on, or to treat what we say with a lot more reverence. So I think therefore requires more safety netting actually, or to give them quite strict parameters about what they should be doing and when. Whereas a young person, I just, I think I feel a lot safer to speak more broadly.”* (GP22, male OOH GP, 6–10 years’ experience).

GPs described two main strategies to safety-netting. Firstly, clinicians would discuss the risk of deterioration with the patient and/or a third party including the rationale for the course of action.

*“If somebody’s at all frail, if they’re at all vulnerable I would probably safety net through a third party, so for example, I might speak to the district nurse or I might speak to a relative who would be able to call in and check and see how they were getting on or speak, with the patient’s permission, speak to a relative about what the safety-netting arrangements are”* (GP19, female partner, ≥21 years’ experience, small rural practice).

Secondly, safety-net plans detailed the conditions where patients should seek medical attention, and granted permission for the patients to contact their doctor. Some GPs felt that this shared responsibility, reduced their clinical anxiety. However, where GPs perceived that patients were less likely to seek help, specific follow up plans were made with the GP (telephone or surgery appointment or home visit) or wider practice team (community visiting nurses).

## Discussion

### Summary

GP participants described the challenge of diagnosing older people with serious infections. Contributing factors included the high levels of multi-morbidity and polypharmacy, atypical presentations, as well as a lack of knowledge of their patients due to a loss of continuity.

Diagnostic challenges were present at each stage of the patient assessment, making recognition of a serious infection difficult. The medical history was frequently disrupted by communication difficulties; the physical examination was not believed to reliably predict the severity of disease in this age group.

Scoring systems were mainly used as communication tools but their validity in the older adult and in community settings was questioned.

Investigations were sometimes used to resolve diagnostic uncertainty, but availability and speed of result limited their practical use. Clear safety-net plans shared with patients and their families helped GPs manage ongoing uncertainty.

### Strengths and limitations

This study used maximum variation sampling to capture the variation and diversity demonstrated within primary care doctors. The authors recognise due to the self-selecting nature of the recruitment strategy, participants may have been particularly interested in the subject; despite this a range of views were demonstrated. The interviewers were both GPs which allowed detailed case discussions, but may have influenced how the participants responded to the interview questions. For example, participants may have assumed the interviewers had a certain level of knowledge which may have limited the data collected.

The older patient with a severe infection is a common clinical scenario in general practice and each participant could recall several relevant clinical cases. However, as the descriptions were drawn from the clinicians’ memory rather than direct observation, hindsight bias is possible [15]. The use of an age cut-off for cases discussed was a pragmatic decision to enable discussion of older patients. However, the authors recognise that age is not synonymous with frailty or poor health.

### Comparison with existing literature

In keeping with previous research, this study has shown the difficulties in the clinical assessment of older people with suspected serious infections. Diagnostic uncertainty is inherent in the process; history taking is complicated by cognitive impairment and confusion [1], physical signs are often absent or altered [2, 16] and the validity of diagnostic tests in this age group is not well characterised [17].

These results also highlight from the GP perspective the impact of losing continuity of care for older patient. Previous work has shown that long-term high-quality primary care relationship is particularly important for this age group [18, 19], with an association between poor continuity and increased emergency department visits and unplanned hospital admissions [18, 20, 21]. This study describes how GPs use this relational continuity to recognise the deteriorating older adult and may explain how continuity improves patient care.

### Implications for practice and research

Continuity is under threat from increasing UK GP workloads [22]. Recent estimates suggest a 27% reduction in perceived relational continuity between 2012 and 2017 [19]. This paper highlights the importance of continuity when diagnosing serious infections in older adults and that maintaining relational continuity must remain a priority for general practice. Suggested strategies to maintain continuity include named GPs, increased patient awareness, alternative appointment booking procedures, and developing community teams coordinating patient care for high risk groups [18] but further research is needed to understand which approaches improve patient outcomes.

Similar to studies in OOH general practice [23], clinical strategies to facilitate shared decision making included safety-netting [24] and risk-based discussions with patients and their kin. There has been increasing interest in how to achieve shared decision making in emergency situations [25] and recent surveys suggest that patients wish to be involved [26]. Further studies are needed to understand patient experience of serious infections and their expectations of shared decision-making in acute care [27].

Point of care testing could be helpful to support GPs make diagnostic decisions. However, as reflected by the experience of our participants, POC tests are currently not in routine use in primary care [28]. The barriers to their use here is threefold: the lack of technological advancement for tests to be fit for purpose in the community, the associated costs, and the lack of evidence base for patient benefit at present [29].

## Conclusion

Diagnostic challenges are present throughout the assessment of an older adult with a serious infection in primary care. Supporting GPs to provide continuity of patient care for older adults may improve the recognition of serious infections and developing point of care testing for use in community settings may reduce diagnostic uncertainty.

## Abbreviations

CAP: Community acquired pneumonia
GP: General practitioner
OOH: Out-of-hours
POC: Point of care
PSI: Pneumonia severity index
